# Solution-Processible Crystalline NiO Nanoparticles for High-Performance Planar Perovskite Photovoltaic Cells

**DOI:** 10.1038/srep30759

**Published:** 2016-07-28

**Authors:** Uisik Kwon, Bong-Gi Kim, Duc Cuong Nguyen, Jong-Hyeon Park, Na Young Ha, Seung-Joo Kim, Seung Hwan Ko, Soonil Lee, Daeho Lee, Hui Joon Park

**Affiliations:** 1 Division of Energy Systems Research, Ajou University, Suwon 16499, Korea; 2 Department of Organic and Nano System Engineering, Konkuk University, Seoul 05029, Korea; 3 Department of Mechanical and Aerospace Engineering, Seoul National University, Seoul 08826, Korea; 4 Department of Mechanical Engineering, Gachon University, Seongnam 13120, Korea; 5 Department of Electrical and Computer Engineering, Ajou University, Suwon 16499, Korea

## Abstract

In this work, we report on solution-based p-i-n-type planar-structured CH_3_NH_3_PbI_3_ perovskite photovoltaic (PV) cells, in which precrystallized NiO nanoparticles (NPs) without post-treatment are used to form a hole transport layer (HTL). X-ray diffraction and high-resolution transmission electron microscopy showed the crystallinity of the NPs, and atomic force microscopy and scanning electron microscopy confirmed the uniform surfaces of the resultant NiO thin film and the subsequent perovskite photoactive layer. Compared to the conventional poly(3,4-ethylenedioxythiophene):poly(styrenesulfonate) (PEDOT:PSS) HTL, the NiO HTL had excellent energy-level alignment with that of CH_3_NH_3_PbI_3_ and improved electron-blocking capability, as analyzed by photoelectron spectroscopy and diode modeling, resulting in *V*_oc_ ~0.13 V higher than conventional PEDOT:PSS-based devices. Consequently, a power conversion efficiency (PCE) of 15.4% with a high fill factor (*FF*, 0.74), short-circuit current density (*J*_sc_, 20.2 mA·cm^−2^), and open circuit voltage (*V*_oc_, 1.04 V) having negligible hysteresis and superior air stability has been achieved.

Recently, organometal trihalide perovskite materials have been investigated extensively for use as light-absorbing material in photovoltaic (PV) cells because of their unique properties such as direct optical bandgap, broadband light absorption that extends to near-infrared (800 nm) with a high extinction coefficient, bipolar transport, and long carrier diffusion length^1^,^2^,^3^,^4^,^5^,^6^,^7^. Furthermore, their excellent compatibility with various organic semiconductors such as 2,2′,7,7′-tetrakis(*N*,*N*-di-*p*-dimethoxyphenyl-amino)-9,9′-spirobifluorene (*spiro*-OMeTAD), phenyl-C_61_-butyric acid methyl ester (PCBM), and conjugated polymers, and inorganic semiconductors such as titanium dioxide (TiO_2_) makes them advantageous for use in various device structures, which have been widely studied for organic photovoltaic (OPV) cells and dye-sensitized solar cells (DSSCs)^1^,^2^,^3^,^4^,^5^,^6^,^7^.

There has been significant improvement in the performance of perovskite-based PV cells since they were first reported^8^, when CH_3_NH_3_PbI_3_ was initially used as a visible-light sensitizer in DSSCs on mesoporous (mp) TiO_2_. The power conversion efficiency (PCE), initially 3.81%, now reaches 20%^9^,^10^. In general, the high-efficiency perovskite PVs have been built on mp metal oxide such as TiO_2_ and Al_2_O_3_, so the use of high-temperature annealing for the mp layer is inevitable^7^,^9^,^10^. Without the mp metal oxide layer, the perovskite photoactive layer can be cast directly onto the continuous buffer layers by forming either n-i-p-type or p-i-n-type planar heterojunction-structured PV cells because of their bipolar property. However, n-i-p-type planar PV cells, which have an electron transport layer such as dense TiO_2_ between the perovskite active layer and the transparent conducting oxide electrode, often have severe hysteretic behavior. This causes variable current-voltage (*I*-*V*) behavior depending on the scan speed, scan direction, and light-soaking time^11^,^12^. Therefore, the high-temperature annealing needed to introduce mp TiO_2_ in high-efficiency PV cells has hampered progress toward low-cost flexible devices that can be fabricated by a high-speed roll-to-roll process.

Though the cause of hysteresis remains in dispute, it is believed to be related to various factors such as ferroelectric polarization, ion migration, trap states, and unbalanced charge transport^9^,^13^,^14^,^15^. Recent reports showed that the hysteretic *I*-*V* characteristics in p-i-n-type planar heterojunction perovskite PV cells having indium tin oxide(ITO)/hole transport layer(HTL)/CH_3_NH_3_PbI_3_/PCBM electron transport layer(ETL)/metal electrode structures were suppressed^16^,^17^. This is beneficial because this structure compensates for the effect of the relatively short hole diffusion length and consequently provides better charge balance, and the PCBM ETL, cast on the photoactive layer, is expected to passivate charge trap states on the surface and grain boundary of the CH_3_NH_3_PbI_3_ perovskite materials^9^,^16^,^17^. Poly(3,4-ethylenedioxythiophene):poly(styrenesulfonate) (PEDOT:PSS) has been widely used as an efficient HTL between the ITO transparent conducting oxide layer and the perovskite photoactive layer in an efficient p-i-n-type planar heterojunction architecture. However, PEDOT:PSS is thought to cause device degradation because it is water-borne and acidic, both of which seriously affect the long-term stability of perovskite PV devices^18^,^19^,^20^.

Nickel oxide (NiO) is a widely used inorganic p-type semiconductor with a large bandgap (*E*_*g*_) and deep valence band (VB) that aligns well with the highest occupied molecular orbital (HOMO) levels of many p-type organic semiconductors. Therefore, its use as an HTL in organic optoelectronic devices such as OPV cells and organic light-emitting diodes (OLED) has been widely studied^21^,^22^. In addition, NiO has been used as an HTL in perovskite-based PV cells. However, most high-performance p-i-n-type perovskite PV cells with NiO as the HTL were fabricated using NiO layer prepared by expensive processes such as pulsed-laser deposition (PLD) and sputtering^23^. For instance, Park *et al*. fabricated perovskite PV cells on nanostructured NiO prepared by PLD that had an average PCE of 15.3% (maximum 17.3%)^24^. Wang *et al*. used NiO_*x*_ film, prepared by sputtering, in perovskite PV cells that had a maximum PCE of 11.6%^25^. For those studies that used solution-based NiO layers, not only was the sol-gel method used to prepare the NiO layer, requiring high-temperature annealing to convert the precursors to NiO or to promote crystallization, but also the PCEs were around 10%^23^,^26^,^27^,^28^,^29^,^30^,^31^,^32^. The solution-based NiO_*x*_ layer of Jen *et al*.^33^ provided an average PCE of 14.98% (maximum 15.40%), but their high-performance NiO_*x*_ layer was doped with Cu. The PCE of their PV cells with pure NiO_*x*_ was 8.73%^33^. Recently, Chen *et al*.^34^ reported an average PCE of 13.5%; however, mp Al_2_O_3_ had been added onto the NiO layer to form a meso-superstructured configuration for better performance. Moreover, high-temperature annealing at 500 °C to promote crystallization of NiO, prepared by spraying, is not applicable for the flexible substrate.

In this article, we present a new approach in which precrystallized NiO nanoparticles (NPs) are used to form an HTL in p-i-n-type planar-structured perovskite PV cells via a simple spin-casting process. Compared to widely utilized conventional PEDOT:PSS-based PV cells, our NiO-based PV devices showed better performance (a maximum PCE of 15.4% with negligible hysteresis) and air stability. We analyzed the characteristics of the NiO layer using photoemission spectroscopy (PES), including X-ray photoelectron spectroscopy (XPS) and ultraviolet photoelectron spectroscopy (UPS), and investigated its morphology using atomic force microscopy (AFM) and scanning electron microscopy (SEM). Furthermore, X-ray diffraction (XRD) and high-resolution transmission electron microscopy (HR-TEM) were used to study its crystallinity. In addition, quantum efficiency and absorbance were used to analyze the performances of the PV cells.

## Results and Discussion

**Figure 1:**
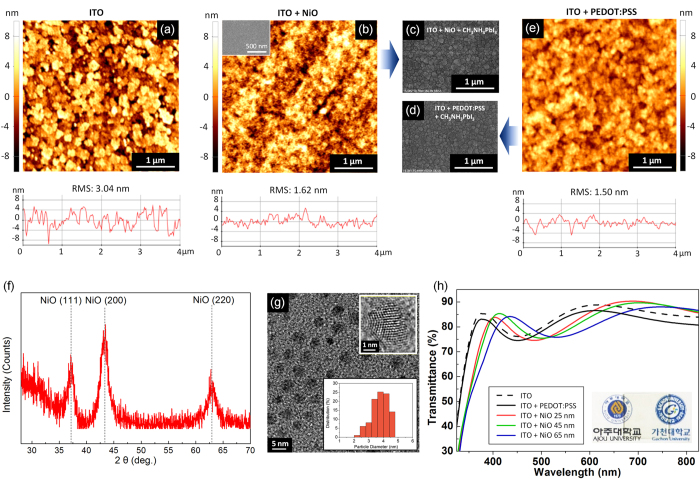
Characteristics of NiO thin film. AFM height images of (**a**) ITO, (**b**) ITO + NiO (45nm) (Inset of (**b**) is SEM image) and (**e**) ITO + PEDOT:PSS. SEM images of (**c**) ITO + NiO (45nm) + CH_3_NH_3_PbI_3_ and (**d**) ITO + PEDOT:PSS + CH_3_NH_3_PbI_3_. (**f**) XRD pattern of NiO thin film. (**g**) TEM and HR-TEM (upper inset) images of crystallized NiO nanoparticles, having interplanar spacing of 0.24 and 0.21 nm that correspond to the (111) and (200) lattice planes, respectively. Bottom inset is the size distribution of NiO NP. (**h**) Transmittance of NiO thin films on ITO according to the thickness of NiO. Inset picture is 45 nm-thick NiO on ITO. The use of university logos in inset of (**h**) was permitted by Ajou University and Gachon University (Copyright 2016, Ajou University and Gachon University).

The NiO NPs utilized in this work to form an HTL between the perovskite photoactive layer and the ITO electrode were synthesized by the reduction of Ni(II) acetylacetonate (C_10_H_14_NiO_4_) via the reducing agent borane-triethylamine complex [(C_2_H_5_)_3_N•BH_3_] in oleylamine (C_18_H_37_N), which was both the solvent and the surfactant. This approach is based on a previously reported method for preparing Ni NPs^35^, but it was notably modified. All of our synthesis procedures could be performed at ambient conditions because the inert gas environment, which was needed in the former report for the synthesis of pure Ni NPs for the catalyst, is not needed for the synthesis of metal oxide NPs. In addition, we demonstrated that commercially available borane-triethylamine complex could be successfully utilized as a reducing agent instead of borane–tributylamine complex, which was used in the former report. Furthermore, we found that the quality of the NiO thin film was affected significantly by residual impurities after synthesis; therefore, the synthesized NPs underwent extra ethanol rinses to thoroughly remove the residual chemicals for better film quality. Moreover, oleic acid, essential for stabilizing Ni NPs for monodisperse NPs as cosurfactants, had no noticeable effect on the performance of the PV cells. We could not find any significant differences between NiO NPs synthesized with and without oleic acid with respect to their characteristics as an HTL in the PV cells. The NiO NP solution was prepared by dispersing the synthesized NPs uniformly in an organic solvent such as tetradecane and then the size of the resultant NPs was determined by TEM to be ~4 nm (Fig. 1g bottom inset).

Uniformity of the HTL film is an important factor because it can directly affect the morphology of following photoactive layer. Unlike in former works^27^,^30^,^32^ in which additional effort was needed to optimize the morphology of the perovskite photoactive layer on the NiO layer because of its nonuniformity, our uniform NiO layer could be prepared on ITO by simply spin-casting NiO NP solution, as shown in the AFM (Fig. 1a,b,e) and SEM images (inset of Fig. 1b). In particular, AFM height images confirmed that the height variation of the NiO surface on ITO was within several nanometers, of which the root mean square (RMS) roughness was 1.62 nm, much lower than that of ITO (3.04 nm) and compatible to that of PEDOT:PSS on ITO (1.50 nm). Therefore, the NiO layer could be a successful alternative to PEDOT:PSS to planarize the rough ITO surface, and probably did not induce any morphological difference in the perovskite photoactive layer compared to that cast on conventional PEDOT:PSS HTL (Fig. 1c,d). This is discussed further below in the discussion on the performance of the PV cells.

Various analytical methods were used to identify the characteristics of the HTL of the perovskite PV cell. First, the crystallinity of the NiO thin film was analyzed by XRD patterns and HR-TEM. The XRD spectrum in Fig. 1f shows strong peaks at 2*θ* = 37.2°, 43.3°, and 62.9°, which correspond to the (111), (200), and (220) planes of a cubic crystal structure. The calculated *d*-spacings are 0.24, 0.21 and 0.15 nm. The spin-cast NiO films had been heated at 270 °C, the boiling point of the tetradecane solvent, for 5 min in air to remove residual solvent. However, use of a much lower temperature probably is feasible if NiO NPs are dispersed into a solvent with a lower boiling point; this is under investigation. The HR-TEM image in the upper inset of Fig. 1g confirms that the synthesized NiO NPs have a cubic crystal structure, of which the interplanar spacings of 0.24 and 0.21 nm correspond to (111) and (200) lattice planes of the cubic rock salt NiO, even without any further treatment such as thermal annealing. Consequently, unlike expensive approaches such as electrodeposition, sputtering, and PLD used to fabricate crystalline NiO layers, our crystallized NiO thin film was prepared using a simple solution-based spin-casting process. Furthermore, different to the conventional solution-based approaches, such as the sol-gel method, in which high-temperature annealing is indispensable to convert precursors to crystallized NiO films, our approach needs marginal heating to remove residual solvent for the crystallized film formation.

**Figure 2:**
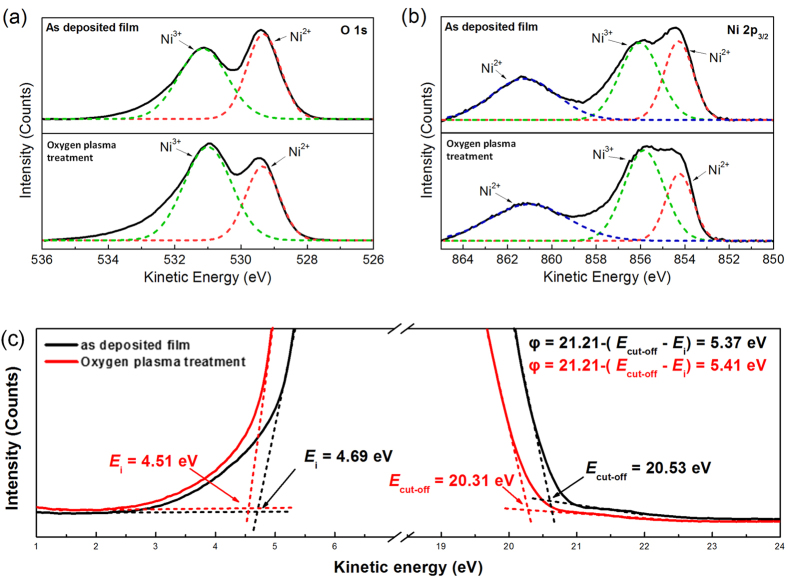
X-ray photoelectron spectroscopy (XPS) results of NiO thin films (pristine and oxygen plasma-treated samples); (**a**) O 1s and (**b**) Ni 2p_3/2_ core level states. (**c**) Ultraviolet photoelectron spectroscopy (UPS) spectra in the onset (*E*_i_) and cut-off (*E*_cut-off_) energy boundary of NiO films before (black line) and after (red line) oxygen plasma treatment.

The electronic structure and composition of the NP-based NiO film were analyzed using XPS. Figure 2a,b show the XPS spectra of O 1s and Ni 2p_3/2_, respectively. The effect of the oxygen plasma treatment on the characteristics of the NiO film was also investigated. The characteristic peaks of the XPS spectra were fitted with Gaussian functions. The Ni 2p_3/2_ peak at 854.3 eV and the O 1s peak at 529.3 eV are from Ni^2+^ and are associated with the Ni─O octahedral bonding of cubic rock salt NiO^36^,^37^,^38^. In addition, the Ni 2p_3/2_ peak at 861 eV is due to a shakeup process in the NiO structure^38^. The Ni 2p_3/2_ peak at 856 eV and the O 1s peak at 531 eV are from metal deficiency Ni^3+^ 37,38, or they also could result from excess oxygen from compounds such as NiOOH^38^,^39^. Ni^3+^ ions, induced by quasilocalized holes around Ni^2+^ vacancies in the lattice, generate p-type conductivity in the NiO_*x*_ thin film^40^,^41^,^42^. Therefore, as shown in Fig. 2a,b, the oxygen plasma-treated NiO layer, having a higher Ni^3+^/Ni^2+^ ratio in the spectra of both O 1s and Ni 2p_3/2_ than the pure NiO layer, is preferred for use as the HTL in PV cells.

**Figure 3:**
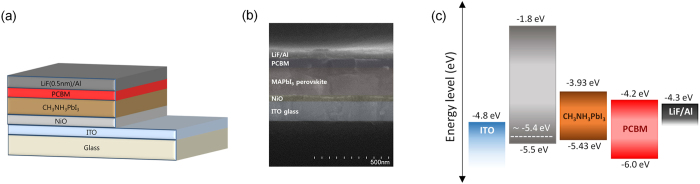
(**a**) The configuration of the perovskite photovoltaic cell. (**b**) Cross-sectional SEM image of device. (**c**) Energy levels for each layer.

The energy band structure of the NiO film was further investigated using UPS. Figure 2c shows the onset (*E*_i_) and cutoff (*E*_cutoff_) energy boundaries in the UPS spectra. The work function (*ϕ*) of the NiO layer, defined as *ϕ* = 21.21 − (*E*_cutoff_ − *E*_i_), was calculated to be 5.37 eV. The configuration of our device (glass/ITO/NiO/CH_3_NH_3_PbI_3_/PCBM/LiF/Al) and the energy levels of each layer^21^,^22^,^23^,^24^,^25^,^26^,^27^,^28^,^29^,^30^,^31^,^32^,^33^,^34^ are depicted in Fig. 3. In this configuration, the calculated work function of the NiO layer aligns well with the energy level of the valence band (VB) of the CH_3_NH_3_PbI_3_ perovskite, which is known to be around 5.43 eV^27^,^31^. Therefore, the NiO layer can be an efficient HTL for charge transport with little energy loss. Furthermore, the work function of the NiO layer slightly increased from 5.37 to 5.41 eV after oxygen plasma treatment, yielding an even better energy level alignment between it and the VB of CH_3_NH_3_PbI_3_ perovskite without additional energy loss.

**Figure 4:**
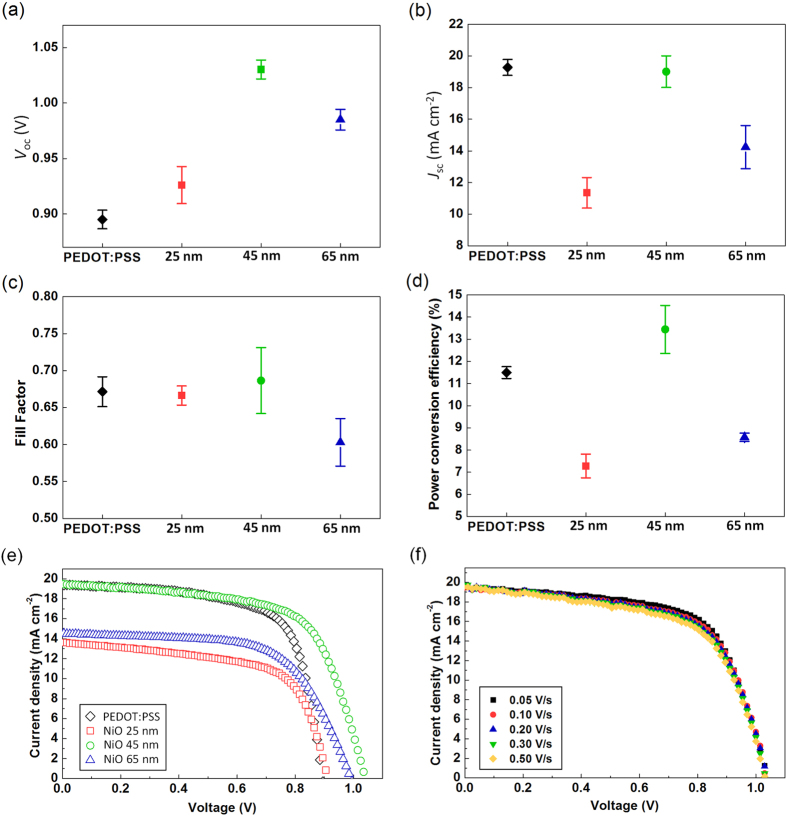
Performances of perovskite photovoltaic cells according to various hole transport layers; (**a**) open circuit voltage (*V*_oc_), (**b**) short circuit current (*J*_sc_), (**c**) fill factor (*FF*), (**d**) power conversion efficiency (PCE), and (**e**) *J*-*V* characteristics. The scale bars in (**a**–**d**) are indicating standard deviations. (**f**) *J*-*V* characteristics of PV cell having 45-nm-thick NiO according to the scan rate in forward direction. All data were measured at AM 1.5 G (100 mW cm^−2^ intensity).

**Figure 5:**
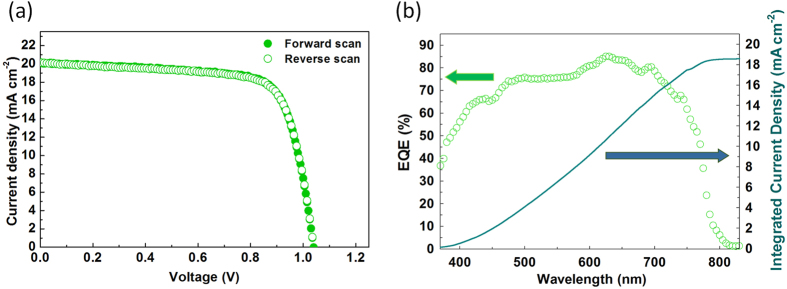
(**a**) *J*-*V* characteristics of the best performing photovoltaic cell, fabricated using 45-nm-thick NiO hole transport layer, according to the scan direction (Forward: *J*_sc_ = 20.2 mA·cm^−2^, *V*_oc_ = 1.04 V, *FF* = 0.74, PCE = 15.4%, Reverse: *J*_sc_ = 20.1 mA·cm^−2^, *V*_oc_ = 1.04 V, *FF* = 0.73, PCE = 15.4%) and (**b**) its incident-photon-to-electron conversion efficiency (external quantum efficiency).

We fabricated perovskite PV cells using NiO as the HTL. The CH_3_NH_3_PbI_3_ perovskite photoactive layer was formed on the oxygen plasma-treated NiO layer on the ITO electrode by spin-casting CH_3_NH_3_PbI_3_ solution in the solvent mixture of γ-butyrolactone and DMSO, along with toluene drop-casting to achieve a uniform perovskite layer^5^. Then, PCBM was added as an electron collection and transport layer on top of the perovskite layer using chlorobenzene, an orthogonal solvent to the perovskite layer. Finally, thermal deposition of LiF and Al completed the fabrication. For comparison, we also fabricated PV cells with PEDOT:PSS as the HTL. The short-circuit current density (*J*_sc_), open-circuit voltage (*V*_oc_), fill factor (*FF*), and PCE of the devices under AM 1.5G simulated sunlight (at 100 mW·cm^−2^ intensity) are summarized in Fig. 4 and Table 1. These parameters were obtained by scanning the *I*-*V* characteristics at a 0.05 V·s^−1^ scan rate. Our NiO-based PV cells were optimized with respect to the thickness of the NiO layer and performed best when the NiO layer was about 45 nm thick, i.e., the average PCE was 13.4% (*J*_sc_ = 19.0 mA·cm^−2^, *V*_oc_ = 1.03 V, *FF* = 0.69), with a maximum PCE of 15.4% (Fig. 5a: *J*_sc_ = 20.2 mA·cm^−2^, *V*_oc_ = 1.04 V, *FF* = 0.74). The corresponding EQE data is shown in Fig. 5b. The PCEs of optimized NiO-based PV cells were even higher than those of widely utilized, conventional PEDOT:PSS-based PV cells that were our controls (average PCE = 11.5%, *J*_sc_ = 19.3 mA·cm^−2^, *V*_oc_ = 0.90 V, *FF* = 0.67). The improved energy-level alignment between the work functions of the HTL and the VB of perovskite that occurred when PEDOT:PSS (*ϕ* = ~5.0 eV) was replaced with NiO (*ϕ* = 5.41 eV for NiO and *ϕ* = ~5.43 eV for VB of perovskite) reduced the potential loss at the interface. Consequently, this induced the noticeable difference between the *V*_oc_ of NiO-based PV cells (average *V*_oc_ of 1.03 V at a thickness of 45 nm) and that of PEDOT:PSS-based control PV cells (average *V*_oc_ of 0.90 V). Moreover, the hysteresis effect with respect to the scan direction (Fig. 5a) and the scan speed (Fig. 4f) was suppressed in our NiO-based PV cells, which indicated that holes and electrons generated in photoactive layer are efficiently transported through NiO HTL and PCBM ETL, respectively, without inducing any charge unbalance.

The variations in performance among the devices having different thickness of NiO layer were further analyzed from a diode point of view using the following generalized Shockley equation^43^:


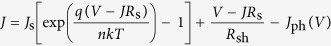


where *R*_sh_ is the shunt resistance, *R*_s_ is the series resistance, *J*_s_ is the dark saturation current density, *n* is the diode ideality factor, and *J*_ph_(*V*) is the voltage-dependent photogenerated current density, which can be obtained by fitting *J*-*V* curves with the equation. The calculated parameters are summarized in Table 1. The thinner NiO layer, 25 nm, had the advantage of a lower *R*_s_ (6.4 Ω·cm^2^), but the decreased electron-blocking property and the increased leakage current caused decreases in *J*_sc_ and *V*_oc_ of devices with the lowest *R*_sh_ (4.46 × 10^2^ Ω·cm^2^). In particular, the uniformity of thin NiO layers was compromised (data not shown), not helpful to have a high *R*_sh_. The thicker NiO layer, 65 nm, had the highest *R*_s_ value (13.3 Ω·cm^2^), which consequently caused the low PCE of the PV cell. Furthermore, the decreased transmittance between 520 and 780 nm (Fig. 1h) directly affected the number of photons absorbed. This could play a crucial role in the lower photocurrent of these devices (65-nm-thick NiO). In addition, the increased *V*_oc_ in the NiO-based PV cells compared to that in PEDOT:PSS-based devices was confirmed by the lower *J*_s_ of the diode, which was evidence of the improved electron-blocking property of NiO-based devices reported previously^24^,^44^. For high *R*_sh_, the ideal diode model is expressed as *V*_oc_ ≈ *nkT*/*q*ln(1 + *J*_sc_/*J*_s_), and NiO-based devices (45 nm thick) had lower *J*_s_ than PEDOT:PSS-based devices; thus, a higher *V*_oc_ was expected from the improved electron-blocking property of the NiO layer. This was supported by the improved *R*_sh_ of NiO-based devices with the optimum 45-nm-thick NiO layer (from 5.11 × 10^2^ Ω·cm^2^ of PEDOT:PSS-based device to 6.31 × 10^2^ Ω·cm^2^). Meanwhile, NiO-based devices and PEDOT:PSS-based devices have similar values for *n*, which is related to charge dissociation in the photoactive layer of the PV cells. This means that the morphology of the perovskite photoactive layer was not affected by the change in HTL, as we mentioned with respect to the uniform surface of our NiO layer.

**Figure 6:**
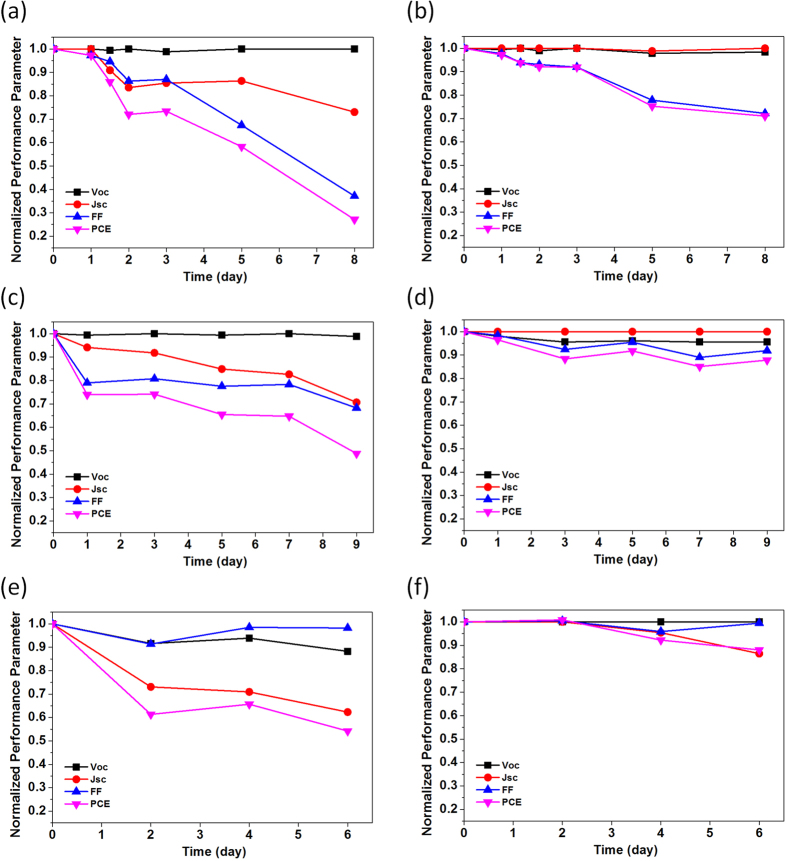
Air storage stability of PV devices with different hole transport layers: (**a,c,e**) PEDOT:PSS, (**b,d,f**) NiO. (**a,b**) PV devices exposed to air (25 °C and 35% relative humidity) without encapsulation. (**c,d**) PV devices stored in N_2_ box without encapsulation. (**e,f**) PV devices fabricated on the hole transport layers, exposed to air (25 °C and 35% relative humidity) during a certain amount of time.

Finally, the air storage stability of NiO HTL-based PV device was investigated (Fig. 6). Because it has been known that the life-time of the PV devices having LiF/Al as an electrode is relatively short due to Li+ diffusion at the interface^45^, 50-nm-thick ZnO layer was added between PCBM and Al electrode instead of LiF, which could be a major source of degradation. Overall device performances were not changed even after replacing LiF with ZnO (data not shown). Figure 6a,b show the performance variations of PEDOT:PSS- and NiO-based PV devices without encapsulation according to the exposure time to air under an ambient condition of 25 °C and 35% relative humidity, respectively. Compared to the PEDOT:PSS-based device showing the fast degradation of PCE to have only 20% of initial performance after 8 days, NiO-based device preserved over 70% of initial PCE. Interestingly, the PCE of PEDOT:PSS-based device, stored in N_2_ box without encapsulation, also decreased to 50% of initial performance, though that of NiO-based device was preserved to 90% of its initial performance, which indicated that the acidic and hygroscopic nature of PEDOT:PSS degraded the device performances even without the exposure to the significant amount of air and moisture. This was further confirmed by fabricating PV devices on the HTLs, exposed to air (25 °C and 35% relative humidity) during a certain amount of time. As shown in Fig. 6e, the PCE of PV device, which was constructed on PEDOT:PSS layer exposed to air for 6 days, showed only 55% of PCE of the PV device, built on fresh PEDOT:PSS layer, and the main performance loss came from the decrease of photocurrent, which may be related to the degradation of the interface between ITO and PEDOT:PSS due to its acidity affecting the hole-collecting property^46^. In contrast, the PCE of PV device, which was constructed on NiO layer exposed to air for 6 days, showed only a small drop of PCE (~90%), compared to that of PV device, prepared on fresh NiO layer, which demonstrated the stability of our NiO layer.

## Conclusions

In summary, we synthesized precrystallized NiO NPs, which had high crystallinity even without additional treatment, to be the HTL in a high-performance perovskite PV cell. The uniform NiO thin film, of which the RMS roughness on ITO was 1.62 nm, was prepared by spin-casting the NP solution. The p-type characteristics of the NiO NPs as an HTL were confirmed by PES. In addition, we showed that the work function of the HTL aligned well with the VB of CH_3_NH_3_PbI_3_ perovskite, which was advantageous for low energy loss in charge transport, and the improved electron-blocking property was related to the decrease in *J*_s_, resulting in *V*_oc_ ~0.13 V higher than that of conventional PEDOT:PSS-based devices. Furthermore, it was shown that both p-type characteristics and the energy-level alignment were improved by the oxygen plasma treatment of the NiO layer. Consequently, a maximum PCE of 15.4% with a high *FF* (0.74), *J*_sc_ (20.2 mA·cm^−2^), and *V*_oc_ (1.04 V) having negligible hysteresis and superior air stability has been achieved.

## Methods

### Synthesis of NiO nanoparticles

Ni(II) acetylacetonate (C_10_H_14_NiO_4_) (1 mmol) was dissolved in 15 ml of oleylamine (C_18_H_37_N) with and without 1 mmol of oleic acid (C_18_H_34_O_2_). The solution was heated to 110 °C for more than 30 min, while vigorously stirred to degas dissolved oxygen and evaporate moisture, and then cooled to and maintained at 90 °C. Next, 2.4 mmol of borane–triethylamine complex [(C_2_H_5_)_3_N∙BH_3_] mixed with 2 ml of oleylamine was injected quickly into the solution. The resultant solution was maintained at 90 °C for 1 h while vigorously stirred and then cooled to room temperature. Ethanol (C_2_H_6_O) (30 ml) was added to the solution and the mixture was centrifuged at 6000–7000 rpm for 15 min to collect the NiO NPs. The NPs were washed with ethanol several times and were dispersed in tetradecane (C_14_H_30_) by ultrasonication. Oleic acid, which was essential for stabilizing Ni NPs for monodisperse NPs as cosurfactants in the literature^35^, had no noticeable effect on the quality of NiO NPs, and we could not find any significant differences between NiO NPs synthesized with and without oleic acid with respect to their characteristics as an HTL in the PV cells. Therefore, we have utilized NiO NP, synthesized without oleic acid having high boiling point at 380 °C, for PV cell fabrication.

### Fabrication of perovskite PV cells

A sequence of acetone, isopropyl alcohol (IPA), and deionized (DI) water was used to clean the patterned ITO-coated glass substrates via ultrasonication. The ITO substrates were treated with oxygen plasma for 5 min before use to improve the wettability of following solution. For the HTL, PEDOT:PSS (Clevios PVP AI 4083) was deposited on the ITO substrate by spin-casting at 3000 rpm for 30 s and annealed using a hot plate at 150 °C for 20 min. For NiO-based PV cells, the NiO layer was formed on the ITO substrate by spin-casting the NP solution instead of PEDOT:PSS. The thickness of the NiO layer was controlled by controlling the concentration of the NP solution and the rotational speed of the spin-casting. For PV cell fabrication, NiO NPs, synthesized without oleic acid, were utilized. The NiO layers were heated at 270 °C, which is near the boiling point of tetradecane, for 5 min to remove residual tetradecane solvent and then slowly cooled. Before casting the perovskite photoabsorber, the NiO layers were treated with oxygen plasma to improve the wettability of following perovskite solution as well as to improve the hole transport property of NiO, as explained in the Results and discussion section. After forming an HTL on the ITO substrate, the samples were transferred to a N_2_ box. CH_3_NH_3_PbI_3_ perovskite solution was prepared by dissolving PbI_2_ (Sigma Aldrich) and CH_3_NH_3_I (1:1 molar ratio), synthesized following the literature^5^,^7^, in the solvent mixture of γ-butyrolactone (GBL) and dimethylsulfoxide (DMSO) (7:3 v/v) for a total concentration of 1.1 M in a N_2_ atmosphere. The solution was stirred at 70 °C for at least 12 h before being used. Then, spin-casting the solution along with toluene drop-casting achieved a uniform perovskite layer, the characteristics of which are described elsewhere^5^. PCBM (20 mg·ml^−1^ in chloroform) was then spin-cast on top of the perovskite layer as an ETL. Both the perovskite photoabsorber and the PCBM layers were prepared in a N_2_ box. Finally, the samples were transferred into a thermal evaporator and LiF (0.5 nm)/Al (80 nm) were thermally deposited at a base pressure of 4 × 10^−6^ torr. As for the PV cells for air storage stability test, 50-nm-thick ZnO layer was added instead of LiF by spin-casting ZnO NP solution (Sigma Aldrich) on PCBM before Al deposition. Overall device performances were not changed after replacing LiF with ZnO. The active area of the PV cell was about 0.06 cm^2^, which was defined by the area where the ITO and Al electrodes overlapped. Over 20 devices were prepared for each process condition.

### Characterization

A solar simulator (PEC-L01, Peccell Technologies, Inc.) with an AM 1.5G filter was used to provide 100 mW·cm^−2^ of illumination on the PV cells, with the intensity calibrated using a Si photodiode. *I*-*V* characteristics were obtained using an Ivium technology Ivium compactstat by scanning the *I*-*V* curves at a 0.05 V·s^−1^ scan rate. The incident-photon-to-electron conversion efficiency (IPCE) was measured under short-circuit conditions using ABET Technology 10500 solar simulator as the light source and a SPECTRO Mmac-200 as the light solution. Ultraviolet–visible absorption spectra were recorded with a Jasco V760 UV-Vis NIR spectrophotometer in the 300–800-nm wavelength range at room temperature. XRD spectra of the prepared films were obtained using a Rigaku Ultima III high-resolution X-ray diffractometer. XPS and UPS (Thermo VG Scientific Sigma Probe) were used for PES. SEM images were obtained by Hitachi S-4800. AFM images were measured by PSIA XE 100.

## Additional Information

**How to cite this article**: Kwon, U. *et al*. Solution-Processible Crystalline NiO Nanoparticles for High-Performance Planar Perovskite Photovoltaic Cells. *Sci. Rep.* **6**, 30759; doi: 10.1038/srep30759 (2016).

## Figures and Tables

**Table 1:** Average values of perovskite photovoltaic cell performances and their diode characteristics according to various hole transport layers.

HTL	Performances				Diode characteristics			
	*J*_sc_ (mA·cm^−2^)	*V*_oc_ (V)	*FF*	PCE (%)	*R*_s_ (Ω·cm^2^)	*R*_sh_ (10^2^ Ω·cm^2^)	*n*	*J*_s_ (10^−8^ mA·cm^−2^)
	Ave. (Std.)	Ave. (Std.)	Ave. (Std.)	Ave. (Std.)				
PEDOT:PSS	19.3 (0.5)	0.90 (0.01)	0.67 (0.02)	11.5 (0.3)	4.5	5.11	1.76	23.8
NiO 25 nm	11.4 (1.0)	0.93 (0.02)	0.67 (0.01)	7.3 (0.5)	6.4	4.46		
NiO 45 nm	19.0 (1.0)	1.03 (0.01)	0.69 (0.04)	13.4 (1.1)	7.0	6.31	1.88	9.47
NiO 65 nm	14.3 (1.4)	0.99 (0.01)	0.60 (0.03)	8.6 (0.2)	13.3	9.16		

*R*_s_: series resistance.

*R*_sh_: shunt resistance.

*n*: diode ideality factor.

*J*_s_: dark saturation current density. Numbers in parentheses are standard deviations.
